# Measuring and assessing motor skills of selected Croatian U12, U14 and U16 tennis players

**DOI:** 10.3389/fphys.2023.1241847

**Published:** 2023-11-30

**Authors:** Petar Barbaros, Bernard Dudašek, Dragan Milanović, Sara Šanjug, Marin Galić

**Affiliations:** ^1^ Faculty of Kinesiology, University of Zagreb, Zagreb, Croatia; ^2^ Faculty of Political Science, University of Zagreb, Zagreb, Croatia

**Keywords:** young tennis players, motor skills, multi-sided physical conditioning, tennis diagnostic, LtAD

## Abstract

**Purpose:** The aim of this research is to analyse and to determine the differences between tennis players in younger age categories (U12, U and U16) in certain motor skills.

**Methods:** A total of 60 tennis players ranked in the rankings of the Croatian Tennis Federation were measured by using 10 tests for assessing explosive strength in jump, speed, agility, and trunk strength. The tennis players were divided into three groups of 20 respondents, depending on the age category in which they compete. Statistically significant differences (*p* < 0.05) between all age categories were found in indicators of frontal and lateral agility, running speed in the 20-m shuttle run test, and explosive strength in jump and repetitive trunk strength.

**Results:** The results of the conducted tests indicate a linear development trend for the mentioned skills in relation with the increase of chronological age of the tennis players. Statistically significantly better results were shown between test subjects under 14 years compared to test subjects under 12 years in tests for the assessment of agility (SST,A9-3-6-3-9), in the 20 m sprint test, in tests of explosive strength of lower extremities (CMJ, CMJmax,SJ) and in the test of repetitive trunk strength (TF). Subjects under 16 years achieved significantly better results compared to subjects under 14 years in tests for assessing agility (SST, A9-3-6-3-9), speed (SRT5m, SRT10m, SRT20m) and explosiveness (CMJ, CMJmax, SJ). Players under 16 years recorded significantly better results in all tests for assessing agility (SST,A9-3-6-3-9), speed (SRT5m, SRT10m, SRT20m), explosiveness of the lower extremities (CMJ, CMJmax, SJ) and in the test for assessing repetitive trunk strength (TF). Statistically significant differences were not detected in tests of running speed in the 5-m and 10-m shuttle run tests among U12 and U14 tennis players, nor between U14 and U16 tennis players in the 60-s trunk flexion test. The highest heterogeneity of results in a single age category was determined in the test for assessing isometric trunk strength, and thus tennis players of different age categories do not differ significantly in this skill.

**Conclusion:** The results of this research point to the development of specific motor skills in accordance with the increase of game demands and chronological age, however, also refer to the problem of muscle imbalance between front and back trunk musculature. Physical conditioning of young tennis players should be multilaterally directed in order to enable injury prevention and adjustment of tennis players to competitive demands.

## 1 Introduction

Tennis belongs to the group of complex polystructural sports which require athletes to have a high level of technical-tactical, physical conditioning and psychological preparation (Dobos and Nagykáldi, 2016; Fett et al., 2017). A high number of specific movement structures and match situations in the tennis game point to the fact that success of tennis players is determined by the level of multiple skills, knowledge, and characteristics. As a result of the afore-mentioned it is difficult to unambiguously determine success factors in the game of tennis. Planning and programming of sports trainings requires good knowledge of competitive demands in specific age categories and competition levels, as well as of player characteristics. Diagnostics of the training level enables detailed insight into the anthropological status of athletes, reveals potential risks of injury and presents the first step in creating an individualized training plan and programme (Ulbricht et al., 2013; Kramer et al., 2017). Regular implementation of diagnostics allows for control of athletes’ development and efficiency of the training process. A detailed insight into the current state of physical conditioning of tennis players is a prerequisite for proper dosage of training and competition load, as well as a “guiding light” in defining objective short-term and long-term goals. Namely, it is precisely the lack of knowledge on the level of development of athletes that is the main cause of applying excessive intensity and load volume, as well as the appearance of overtraining, which often results in premature termination of playing competitive tennis (Strand and Samuelson, 2021).

Lately we have witnessed an increasingly rapid development of the tennis game. Technical-tactical preparedness of tennis players in top-level tennis is at a high level, thus without an optimal level of physical conditioning and movement technique tennis players are unable to use their full potential and be competent at the highest competition levels. Physical conditioning has a major impact on tennis performance even among younger age categories and presents one of the factors for predicting competitive success (Kovacs, 2007; Reid and Schneiker, 2008; Girard and Millet, 2009; Fett et al., 2017), and as a result of the aforementioned, and is taking on an increasing relevance in integral physical conditioning of young tennis players. Researches have shown that physical conditioning abilities in pre-puberty (up to the age of 12) are to the smallest extent in correlation with competitive success (Kovacs, 2007; Ulbricht et al., 2016; Kramer et al., 2017). In fact, in the mentioned period, success depends on the technical performance of strokes, as well as their efficiency and precision. After the age of 12, an accelerated physical growth and development takes place, and an increase in physical height and muscle mass occurs, thus physical conditioning becomes one of the factors which distinguish between successful and less successful tennis players.

Already in younger age categories, tennis players must be prepared to endure a high training load. Technical-tactical trainings for perspective junior players should be implemented in a fund of 15–20 h per week (Reid et al., 2007; Ulbricht et al., 2013) in order to allow them to achieve a high level of play and to participate in major competitions. It is thus clear that very little time remains for implementing physical conditioning trainings, and therefore it should be carried out in the most efficient manner, and tailored in accordance with the athlete’s characteristics.

Specific characteristics of tennis performance should also be taken into consideration in the process of creating battery of tests for assessing motor and functional abilities of tennis players in order to meet the ecological validity of the tests. The aforementioned refers to the level of correspondence between real game situations in which the respondents manifest a certain ability with the situation in which the testing is conducted.

Movement in tennis is characterized by explosive starting velocities, short distance sprints, accelerations and decelerations, changes of direction of movement and performing strokes from various balance positions. It is precisely well-developed motor and functional abilities in flexibility, coordination, vigour and endurance, strength, agility, and speed that enable tennis players aged 12, 14, 16 to overcome different game situations on the tennis court in a strong, fast, long-lasting, precise or coordinated manner.

In this paper, motor skills of tennis players in the U12, U14, and U16 age categories were evaluated by using tests for assessing agility, explosive strength in speed, explosive strength in jump, as well as tests for assessing relative repetitive and static trunk strength.

Reactive agility, that is change of direction of movement conditioned by a reaction to visual stimulus, comes to the fore in the tennis game (Sheppard and Young, 2006; Young and Farrow, 2013). It allows the tennis player to reach the ball in time and to optimally set up for the stroke. Other motor skills, such as coordination, explosive strength, and speed, also have an impact on agility, however, likewise does the technique of changing the direction of movement, as well as perception of the environment and decision-making speed (Sheppard and Young, 2006).

In addition, tennis also demands explosive movements of the entire body. Explosive first steps enable tennis players to quickly arrive at an optimal position for the stroke, as well as allow for lower time-space pressure for playing the stroke. Both muscles of the upper and lower body are active in tennis and their synergy and timely activation of certain segments of the body allow for performing a biomechanically efficient stroke. Explosive strength of the arms and shoulder girdle allow the tennis player to accelerate with the racquet towards the point of contact with the ball, which thus results in a more powerful stroke. Whereas repetitive strength enables performing multiple repetitions of various movement structures and strokes over a longer period in the duration of a match.

The ability of speed is of great importance in the tennis game, as it allows tennis players to arrive at the ball in a timely manner, as well as to quickly perform certain tennis elements. It should be mentioned that there is also specific speed in tennis which is manifested in the ability to performtechnical-tactical elements or movements on the tennis court in the shortest possible time (Kovacs, 2009).

Several studies (Girard and Millet, 2009; Ulbricht et al., 2013; Lambrich and Muehlbauer, 2022) were conducted with the aim to identify the most important characteristics which determine competitive success in junior tennis players. Some of them indicate that motor and functional abilities do not enable predicting competitive success in younger age categories. Whereas other studies indicate that specific abilities and characteristics of younger tennis players, such as agility, speed, and vertical jump, are in correlation with competitive success. The systematic review and meta-analysis by Lambrich and Muehlbauer (2022) examined the impact of competition levels on physical fitness and stroke performance in tennis players, differentiating between elite and sub-elite players. The results indicated clear advantages in physical fitness and stroke performance among elite players, particularly in terms of lower extremity muscle power, endurance, and agility. These findings emphasize the need to design targeted training programs, especially for sub-elite tennis players, to improve these essential physical attributes.

It is important to mention that younger age categories demand a professional and quality approach in planning and programming of the training process. Namely, young male and female tennis players are in a turbulent period of accelerated growth and development. It is precisely in that period that by means of a controlled and individualized training approach that the preconditions for a high level of playing tennis at a senior age are created. The starting point of any training process is diagnostics and analysis of the current training level of a tennis player. This provides insight into the level of development of individual abilities of a tennis player, and thus on the basis of comparison with modal values, provides guidelines for future planning and programming of the training process. The aim of this study is to determine the differences between groups of tennis players in younger age categories (U12, U14 and U16) in motor skills as indicators of physical conditioning.

## 2 Materials and methods

### 2.1 Sample of respondents

The sample of respondents is made of 60 Croatian male tennis players who are ranked in the official rankings of the Croatian Tennis Federation. The respondents were distributed into three groups of 20 tennis players according to their age category, as shown in Table 1. The inclusion criteria for this study involved selecting participants who were active tennis players regularly engaged in training. The participants needed to be within the specified age categories (U12, U14, and U16), aligning with the research objectives. Also, Selected participants were individuals who engaged in tennis training at least three times per week. Exclusion criteria included the exclusion of individuals with serious injuries that could affect their physical performance at the start of the study. This careful selection process aimed to ensure that the participants met the necessary requirements to contribute meaningfully to the research. Also, participants with a medical history or conditions that could potentially influence their physical abilities during the study were excluded. This criterion aimed to maintain the integrity of the results by ensuring that participants’ performance was not influenced by underlying health factors. The recruitment process involved contacting and selecting tennis players who were part of the rankings administered by the Croatian Tennis Federation. These players were already engaged in competitive tennis, which made them suitable candidates for this investigation into motor skill differences among age categories in the sport.

**TABLE 1:** Descriptive indicators of subject characteristics.

age category	n	Age	Height	Body mass
U12	20	12.12 ± 0.43	157.79 ± 8.49	45.07 ± 7.68
U14	20	13.95 ± 0.63	170.43 ± 9.86	56.56 ± 10.22
U16	20	15.89 ± 0.42	178.86 ± 7.79	66.85 ± 7.38

### 2.2 Sample of variables

The respondents were tested by using a total of 10 tests for assessing motor skills, divided into 4 groups. The first group was made of tests for assessing the agility of tennis players: agility test with turn 93,639 (A9-3-6-3-9) and lateral side-step test (SST). The second group of variables was used for assessing explosive strength in sprint by means of the following tests: 5-m shuttle run test (5mSRT), 10-m shuttle run test (10mSRT) and 20-m shuttle run test (20mSRT). In the third group of variables, explosive strength in jump was evaluated with the following tests: countermovement jump (CMJ), countermovement jump–arm swing (CMJmax) and squat jump (SJ). The fourth group was composed of tests for assessing trunk strength: 60-s trunk flexion (TF) and static back-extension endurance test (BE).

### 2.3 Measurement protocol and study design

Measurements were conducted by educated measurers at the premises of the Sports Diagnostics Centre at the Faculty of Kinesiology University of Zagreb. All of the respondents were informed on the purpose and aim of the research, and they participated in the study with the consent of their parents/legal guardians. Before the testing session, all participants completed a standardized warm-up specific to tennis. The warm-up consisted of various activities, including light-intensity running covering a distance of 10 × 20 m. Following the running component, participants engaged in dynamic stretching exercises for a total duration of 15 min. These dynamic stretches involved lateral movements, skipping, jumping, lunges, and concluding with four repetitions of sub-maximum acceleration. Subsequently, the order of the various tests was predetermined and standardized. First, the participants underwent tests to assess agility, followed by tests for explosive strength in sprint, and finally, explosive strength in jump. After the final performance of the tests, all participants had an additional 5-min cool-down period, which consisted of light jogging and static stretching exercises to gradually reduce heart rate and promote muscular recovery. This structured warm-up and cool-down routine ensured that all participants were physically prepared for the study’s assessments and that their physical condition returned to baseline.

Agility Test with Turn (A9-3-6-3-9): This test evaluates agility through a sequence of 9 steps forward, 3 steps backward, 6 steps forward, 3 steps backward, and 9 steps forward. The sequence challenges players’ agility, footwork, and coordination. It comprises three trials with a 30-s rest between each trial and is conducted on a standard tennis court with no specific materials required.

Lateral Side-Step Test (SST): The SST assesses lateral agility as players side-step quickly to the left and right. It focuses on the ability to change direction rapidly, a crucial skill in tennis. The test includes three trials with a 30-s rest between each trial and is conducted on a standard tennis court, requiring no special materials.

5-m Shuttle Run Test (5mSRT): Players sprint back and forth over a 5-m distance in the 5mSRT, measuring their speed and quick acceleration. It comprises three trials with a 30-s rest between each trial and is conducted on a flat, non-slip surface.

10-m Shuttle Run Test (10mSRT): Similar to the 5mSRT, the 10mSRT evaluates players’ speed and acceleration but over a longer 10-m distance. It includes three trials with a 30-s rest between trials and is performed on a flat surface.

20-m Shuttle Run Test (20mSRT): The 20mSRT assesses speed and endurance as players shuttle back and forth over a 20-m distance, necessitating sustained sprinting. The test comprises three trials with a 30-s rest between each trial.

Countermovement Jump (CMJ): In the CMJ test, players perform a vertical jump starting from a standing position. It assesses their explosive leg power. It includes three trials with a 30-s rest period between each trial.

Countermovement Jump–Arm Swing (CMJmax): Similar to the CMJ, the CMJmax test adds an arm swing to maximize vertical jump height. It further evaluates leg power with an emphasis on coordination. It comprises three trials with a 30-s rest between trials.

Squat Jump (SJ): The SJ test requires players to jump vertically from a squatting position, focusing on their leg power, coordination, and technique. It consists of three trials with a 30-s rest between each trial.

60-Second Trunk Flexion (TF): The TF test measures the endurance of the trunk flexor muscles as players perform continuous trunk flexion movements for 60 s. It is conducted on a flat surface, with no specific materials needed.

Static Back-Extension Endurance Test (BE): In the BE, players maintain a static back-extension position to evaluate the endurance of their lower back muscles. While the text does not specify the number of trials or rest periods for this test, it is performed on a flat surface without the need for additional materials.

### 2.4 Data processing methods

Data processing and statistical analysis was performed in the Statistica programme v14.0.0. For all of the variables parameters of descriptive statistics were calculated: arithmetic mean (AM), standard deviation (SD), as well as the measures of asymmetry and distortion of distribution - skewness (Skew) and kurtosis (Kurt). The Cohen’s d coefficient as an indicator of effect size was calculated. Thresholds for effect size were statistically set to the following parameters: insignificant (<0.20), small (0.20–0.50), medium (0.50–0.80), and large (>0.80).

For determining statistically significant differences between the 3 groups of respondents in the measured variables, the ANOVA–univariant analysis of variance was used, as well as the Bonferroni Post-hoc method for analysis of differences, with the level of statistical difference of *p* < 0.05.

## 3 Results

### 3.1 Descriptive indicators of all variables and differences between age categories

#### 3.1.1 Differences between tennis players in U12, U14 and U16 age categories in tests for assessing motor skills

In tests for lateral side steps (SST) and 93639 sprint with turn (A9-3-6-3-9) statistically significant differences were registered in all age categories, thus between U12 and U14, between U14 and U16, and between U12 and U16. It should be noted that there is a continuous improvement in the results of the aforementioned tests with the increase of chronological age of the respondents (A9-3-6-3-9 (AM) = 9.3117 s/8.6933 s/8.1481 s; SST (AM) = 10.130 s/9.058 s/8.2810 s), that is, the respondents require less and less time to complete the chosen test.

The obtained results show that all age categories statistically significantly differ in the 20-m shuttle run test (20mSRT (AM) = 4.12 s/3.89 s/3.56 s), as well as that with the increase of chronological age there is also an improvement of results for the 20-m shuttle run test. Upon analysis of the passing times at 5 m and 10 m, it is evident there are statistically significant differences as well among all age categories, except between U12 and U14 191 tennis players (5mSRT (AM) = 1.6833 s/1.6290 s/1.5190 s; 10mSRT (AM) = 2.5570,192 s/2.4537 s/2.2860 s).

Statistically significant differences were indicated between all age categories in the performance of the countermovement jump (CMJ (AM) = 32.603 cm/36.583 cm/43.795 cm), the countermovement jump with arm swing (CMJmax (AM) = 38.307 cm/44.365 cm/52.543 cm) and the squat jump (SJ (AM) = 31.067 cm/35.398 cm/41.710 cm). In all three of the mentioned variables, a linear progression of results was noted. Furthermore, the differences between the countermovement jump and the countermovement jump with arm swing were the greatest in the U16. age category, while it was the smallest in the U12 age category. The results achieved in the squat jump test were lower than the results of the countermovement jump in all of the groups of respondents, which is to be expected considering the method of performing in individual tests.

From the results achieved in the test for assessing repetitive trunk strength it is evident that there are statistically significant differences between all age categories, except between U14 and U16 tennis players (TF (AM) = 46.650 reps/56.200 reps/59.150 reps). There was no statistically significant difference found between the age categories in the test for assessing static trunk strength (BE 207 (AM) = 105.30 s/120.35 s/119.28 s).

Table 1 showes basic descriptive parameters of measured variables for assessing motor skills of tennis players in U12, U14 and U16 age categories. Furthermore, Table 2 shows high result dispersion in the trunk extension test, where some. respondents achieved significantly below-average results, whereas certain respondents had significantly above-average results.

**TABLE 2:** Basic descriptive parameters (AM, SD, p) and statistical significance of differences (p) and effect size (ES) between respondents in different age categories of measured variables.

Variables	U12	U14	U16	p; ES	p; ES	p; ES (U12:U16)
	AM ± SD	AM ± SD	AM ± SD	(U12:U14)	(U14:U16)	
A9-3-6-3-9	9.31 ± 0.63	8.69 ± 0.42	8.15 ± 0.49	0.001; 1.15	0.005; 1.18	0.001; 2.06
SST	10.13 ± 0.89	9.06 ± 0.67	8.28 ± 0.37	0.001; 1.36	0.002; 1.44	0.001; 2.71
5mSRT	1.68 ± 0.13	1.63 ± 0.11	1.52 ± 0.11	0.441; 0.42	0.013; 1.00	0.001; 1.33
10mSRT	2.56 ± 0.17	2.45 ± 0.14	2.29 ± 0.11	0.081; 0.71	0.002; 1.27	0.001; 1.89
20mSRT	4.12 ± 0.23	3.89 ± 0.17	3.56 ± 0.16	0.001; 1.14	0.001; 1.99	0.001; 2.82
CMJ	32.60 ± 4.13	36.58 ± 4.77	43.80 ± 4.52	0.020; 0.89	0.001; 1.56	0.001; 2,59
CMJmax	38.31 ± 4.22	44.37 ± 8.38	52.54 ± 4.29	0.006; 0.91	0.001; 1.22	0.001; 3.34
SJ	31.07 ± 3.86	35.40 ± 4.12	41.71 ± 3.91	0.003; 1.08	0.001; 1.57	0.001; 2.73
TF	46.65 ± 6.89	56.20 ± 4.55	59.15 ± 6.63	0.000; 1.64	0.398; 0.52	0.001; 1.85
BE	105.30 ± 36.65	120.35 ± 41.57	119.28 ± 40.17	0.700; 0.38	1.000; 0.03	0.803; 0.36

^*^Level of significance *p* < 0.05.

## 4 Discussion

The conducted research showed statistically significant differences in the majority of the observed motor skills between tennis players in the U12, U14, and U16 age categories. Consequently, our assumption that such differences would be present has been confirmed. These results will be discussed in comparison with existing literature in the following text.

The obtained results point to a trend of development of certain motor skills with the increase in chronological age of tennis players. It should be noted that there is a linear increase in the observed abilities with the transition to a higher age category. In fact, the increase in chronological age is also followed by an increase of competitive demands, and thus physical conditioning of tennis players should be at an increasingly higher level. The development of individual abilities is a result of repeating training requirements and specific movement structures within tennis trainings and competitions, however, also of individual physical conditioning trainings. The observed age categories of respondents should be taken into account, as well as the fact that with entering into puberty and the accelerated growth and development phase physical conditioning becomes one of the factors which contributes to competitive success. It is precisely in this period that there is also an increase in longitudinal and transversal dimensionality of the skeleton, which is accompanied by an increase of muscle mass, and that all of the aforementioned has a positive effect on the development of certain motor skills (Kovacs, 2007; Dobos and Nagykáldi, 2016; Ulbricht et al., 2016; Kramer et al., 2017). Technical-tactical preparation and development of players in younger age categories is of crucial importance, however, multi-sided physical conditioning also enables players to keep up with training and competitive demands, as well as optimal tennis performance, and thus presents an indispensable component of a long-term training plan and programme.

### 4.1 Differences between tennis players in U12, U14 and U16 age categories in certain motor skills

#### 4.1.1 Differences between tennis players in U12, U14 and U16 age categories in agility

Tennis movement is characterized by explosive accelerations and decelerations in short distances, as well as constant changes of direction of movement. Due to the aforementioned, lower-extremity explosive strength, starting acceleration and agility play an important role in physical conditioning of tennis players of all age categories (Reid and Schneiker, 2008; Munivrana et al., 2015; Dobos and Nagykáldi, 2016). Numerous research (Fernandez et al., 2006; Filipčić et al., 2010; Munivrana et al., 2015; Kramer et al., 2017; Galé-Ansodi et al., 2016; Fernandez-Fernandez et al., 2010) show a high level of correlation between agility and explosive strength in jump with competitive success and ranking position of tennis players in younger age categories. In the conducted research, statistically significant differences were determined between all age categories in tests of frontal and lateral agility. The results demonstrate a linear progression of this ability with chronological age of tennis players.

Agility is most often developed in individual physical conditioning trainings, however also within tennis trainings and appearances in numerous competitions. Since the process of maturation also results in an increase of longitudinal dimensionality of the skeleton and an increase of muscle mass, the aforementioned positively affects the strength of lower extremities and the speed of movement, and therefore agility as well. The results of this test depend on multiple abilities and motor skills of tennis players, to which attention should be paid in the development of agility. Tennis players with more efficient technique in changing the direction of movement, who are more explosive, and have greater eccentric strength of lower extremities, allowing them to decelerate more efficiently, shall also achieve better results in the agility assessment test (Sheppard and Young, 2006; Sekulic et al., 2017; Keller et al., 2020). In tennis we are referring to reactive agility because during the match a tennis player must quickly react to situations during a point, and accordingly, change positions on the tennis court. The aforementioned shows that by means of the conducted tests assessment is made of pre-planned agility due to the absence of external stimuli, which certainly does not comply with the demands of the tennis game. In order for the testing to come closer to real conditions on the tennis court, agility assessment tests should also include a component of reaction to external stimuli. This is precisely why certain authors proposed a standardized agility test that aside from the change of direction of movement also includes a cognitive component (perception), as well as specific performance of a task with the tennis racquet (Fernandez-Fernandez et al., 2014). In agility training, as well as in other abilities relevant for success in tennis, the transfer of abilities to specific conditions on the tennis court should also be taken into consideration. For this reason, the mentioned ability should be trained in short distances on the tennis court, with focus on lateral and frontal movements, and with connecting imitations or playing strokes.

#### 4.1.2 Differences between tennis players in U12, U14 and U16 age categories in straight sprint speed ability

The increasing demands of the tennis game with transition to a higher age category must likewise be followed by an increase in training stimuli in order to reduce the risk of injury, as well as to improve performance on the court. A tennis player’s speed plays an important role in predicting competitive success. An explosive first step and the ability of starting acceleration are required to efficiently perform strokes from various positions on the tennis court. Tennis players aged between 11 and 13 are capable of performing serves at the speed of approximately 125 km/h, which means that the player returning the serve has 0.69 s for perception, reaction, arrival at the ball, and preparing the stroke (Ferrauti and Bastiaens, 2007). Even small differences in running speed at 5 m can result in a significant advantage or disadvantage in the game. In fact, if a tennis player fails in taking an optimal position for the stroke, the efficiency of the stroke significantly reduces. Stroke velocities in younger age categories are quite high, and as court dimensions are relatively small (8.23 × 23.77 m), speed of movement in short distances in various directions represents one of the crucial success factors. Statistically significant differences in the test for passing time results at 5 and 10 m were not found between U12 and U14 tennis players. The reason for the aforementioned can be in the stagnation of strength development of lower extremity musculature between these two age categories, which is in correlation with the fact that tennis players have not yet reached their peak height velocity (PHV), when a significant increase in muscle mass also takes place. Since the result of the mentioned tests also depends on movement technique, acceleration, and the speed of starting reaction, it is possible that insufficient attention has been given to this segment of physical conditioning. The mentioned results should be discussed, as well as studies should be conducted in the direction that shall show the reasons for such results in the aforementioned parameter, in order for physical conditioning coaches to have the insight for programming trainings in the future for the development of the specific segment of performance which is key for such results. As tennis players run an average of 3–4 m between two strokes, and since they are not able to achieve maximum speed of movement (which occurs between 30 and 60 m in straight-line running) (Fernandez-Fernandez et al., 2014; Dobos and Nagykáldi, 2016), it is precisely the ability of accelerating and stopping at a short distance that is of key importance for tennis players. The passing time at 5 m provides insight into the speed of starting reaction and the first step, while the passing time at 10 m measures the acceleration of an athlete. Starting velocity and acceleration are specific for tennis demands, and therefore precisely the mentioned two tests are of key importance for assessing the specific speed of tennis players.

It is interesting that statistically significant differences were found between all age categories in the 20mSRT test. The mentioned test serves for evaluating maximum running speed, and thus it is not specific for tennis because tennis players do not run a 20-m straight line distance in any single game situation. The obtained results point to the fact that there is a noted increase in the running speed of tennis players between the U12 and U14 age categories, however that there is no progress in efficient starting and acceleration.

#### 4.1.3 Differences between tennis players in U12, U14 and U16 age categories in explosive strength in jump ability

Explosive strength of the lower extremities is assessed by using different types of vertical and horizontal jumps. The vertical jump is a frequently present movement structure in most sports. In terms of movement biomechanics, a similar movement structure also occurs in acceleration, as well as in dynamic game situations (Fernandez-Fernandez et al., 2014). The results of tests for assessing explosive strength in jump demonstrate a linear progression in the mentioned ability with the increase in age category. Statistically significant differences between all age categories indicate a positive effect of growth and development, specific training, and the increase of muscle mass in lower extremities on explosive strength of the lower extremities. Better results with the increase of chronological age can be correlated with higher activation of motor units, better technique of movement performance and improved inter- and intra-muscular coordination (Munivrana et al., 2015). The mentioned ability is of great importance for success in tennis. It enables tennis players to have explosive starts and starting acceleration, it positively affects sprinting speed and agility, as well as participates in the performance of all strokes, as it is precisely the lower extremities which are the first link of the kinetic chain during the performance of all strokes (Dobos and Nagykáldi, 2016; Kramer et al., 2017).

The conclusion can be made that starting speed improves with the development of the mentioned motor ability, which can result with dominance in certain parameters that separate an average and a top-level player. The aforementioned is particularly important because of the relevance of explosive movements and accelerations which allow tennis players to arrive at the ball in a timely manner and to perform strokes from an optimal balance position. Tennis players who are capable of producing a large amount of force in the shortest possible time shall be able to move quickly on the court and perform strokes at high speeds. Improving explosive strength of the lower extremities is important in younger age categories as it allows the players to perform explosive starting acceleration more efficiently, as well as to accelerate in short distances and to produce a larger impulse of force which is thus transferred through other links of the kinetic chain into contact of the racquet with the ball.

The countermovement jump is closest by its performance characteristics to the specific musculature working regime of the lower extremities during tennis performance. Namely, the mentioned test evaluates explosive strength of elastic character, as after the eccentric phase and storage of elastic energy, it is then directed into the concentric phase of the jump. The described working regime is characteristic for the performance of all strokes in tennis, and it is perhaps the most visible during the performance of the serve.

#### 4.1.4 Differences between tennis players in U12, U14 and U16 age categories in repetitive and static trunkstrength

Already in younger age categories, tennis players are exposed to high levels of stress on the locomotor system due to training and competitive load. As tennis includes repeated movements which dominantly activate one side of the body, muscle imbalance and risk of injury as a result ofoverexertion frequently occur. Strength and muscle endurance trainings should be included in the training plan and programme of young tennis players in order to improve the quality of stroke performance, as well as to reduce risk of injuries. A significant ncrease of muscle mass and strength is noted immediately after the period of PHV, which occurs around the age of 14 among boys (Dobos and Nagykáldi, 2016; Kramer et al., 2016; Ulbricht et al., 2016; Kramer et al., 2017).

Statistically significant differences between age categories were found in indicators of repetitive trunk strength, however not for static strength. A well-trained trunk musculature allows for adequate trunk stability during the performance of all strokes, which reduces risk of injury, while it increases stroke control and precision. Furthermore, the trunk represents the central part of the kinetic chain during the performance of strokes, and it is precisely the trunk that is the central link through which energy is transferred from the lower towards the upper extremities (Filipčić et al., 2010; Kovacs and Ellenbecker, 2011; Söğüt, 2016; Myers and Kibler, 2018). The large dispersion of results in the trunk extension test serves as a warning for neglecting the development of static trunk strength among tennis players in younger age categories. Since there were no differences found between the age categories, it is considered necessary to determine the reasons for this unsatisfactory trend. Due to the aforementioned results which show a certain imbalance in the level of development between the front and back side of the trunk, preventive and corrective exercises should be applied in order to reduce the possibility of injuries. The relevance of trunk strength is significantly demonstrated in the serve stroke where the trunk muscles present a key factor for the quality of performance. An optimal level of strength in all muscle-joint systems is very important, however, particular emphasis should be awarded to the muscles of the rotator cuff, forearm, wrist, lumbar part of the back and the trunk due to an increased load on the mentioned parts of the body (Strand and Samuelson, 2021). It should be discussed how much of an effect on the quality of the serve, and also of other strokes, does an insufficient static trunk strength have in younger age categories.

Exercises of concentric and eccentric working regime should be included into training contents for the development of strength, as it is shown that both result in an increase of stroke efficiency and speed (Kovacs, 2007). Furthermore, the aforementioned also reduces muscle imbalance and the possibility of injuries. The most efficient strength training in younger age categories is training in dynamic conditions by using multi-joint exercises with progressive increase of external load, and with emphasis on performance technique and similarity of movements to the technique of movement structures and strokes in tennis (Munivrana et al., 2015).

### 4.2 Strengths and limitations

Strengths of this study include its systematic and comprehensive approach to examining differences in physical fitness and stroke performance in tennis players across competition levels. The systematic review and meta-analysis considered a substantial number of studies and provided a quantified analysis of the differences, which enhances the robustness of the findings. Additionally, this research contributes to our understanding of competition-level differences and provides valuable insights into the physical attributes that distinguish elite and sub-elite tennis players. This study has certain limitations that should be considered. Firstly, the motor tests were conducted with a convenient sample of participants under controlled conditions. While this sample size is reasonable for a study of this nature, it might not fully represent the entire population of tennis players, and results may vary with a larger, more diverse sample. Secondly, the study focuses on tennis players within specific age categories (U12, U14, and U16). The findings may not be directly applicable to older or younger players or those in different competitive environments. Thirdly, a longitudinal study would be needed for a more comprehensive understanding. Also, the study employed specific tests to assess motor skills, and the choice of tests could impact the results. In future studies, it is crucial to acknowledge that different tests may yield varying outcomes, introducing the potential for bias based on test selection. Therefore, researchers should carefully consider the choice of tests to ensure a well-rounded evaluation of tennis players’ abilities. Furthermore, future research endeavors could greatly benefit from continuous monitoring of the correlation between the development of motor and functional abilities, not only in male but also female tennis players, and other components of an individual’s anthropological status in relation to competitive success within younger age categories. Particular attention should be given to selecting tests that closely replicate the demands of the tennis game, allowing for a consistent insight into the normative values of tennis players’ abilities and characteristics that significantly contribute to success within specific age categories. Additionally, future studies could incorporate longitudinal monitoring of the anthropological status of both male and female tennis players, which would not only shed light on differences between age categories but also on the individual development trajectory of each player. These comprehensive research directions will provide invaluable insights into the intricate relationship between physical development, athletic performance, and competitive success in the realm of tennis. This would enable a more detailed view on the effect of age, maturation, and the training process on the anthropological status of tennis players. The aforementioned approach would offer a detailed insight into the development of significant abilities and characteristics of athletes, as well as allow for corrections of the training plan and programme.

## 5 Conclusions

The results obtained in this research indicate a linear progression trend in the development of most of the evaluated motor skills with the increase of age category of tennis players. Based on the results of this research, we can confirm our assumption that younger tennis players in the U12 category may exhibit significant differences in certain motor skills compared to those in the U14 and U16 categories. The observed age categories are of great importance as tennis players are at the beginning of their careers and in a phase of intensive growth and development. An individualized plan and programme, based on diagnostics, enables maximal use of a player’s potential and allows for potentially achieving a successful long-term sports career.

## Data Availability

The raw data supporting the conclusions of this article will be made available by the authors, without undue reservation.
